# Supplementary material for: FOXO/DAF-16 modulates the transcription factor ROR/NHR-23 and inhibits the let-7 microRNA to maintain multipotency during dauer
Source: bioRxiv. 2026 Jul 20:2026.07.18.739353. Preprint. [Version 1] doi: 10.64898/2026.07.18.739353 (PMC13419435; doi:10.64898/2026.07.18.739353)
Supplement: Supplement 2 [file NIHPP2026.07.18.739353v1-supplement-2.pdf]

bioRxiv preprint doi: https://doi.org/10.64898/2026.07.18.739353; this version posted July 20, 2026. The copyright holder for this preprint (which was not certified by peer review) is the author/funder, who has granted bioRxiv a license to display the preprint in perpetuity. It is made available under a CC-BY 4.0 International license.

Figures

Figure S1

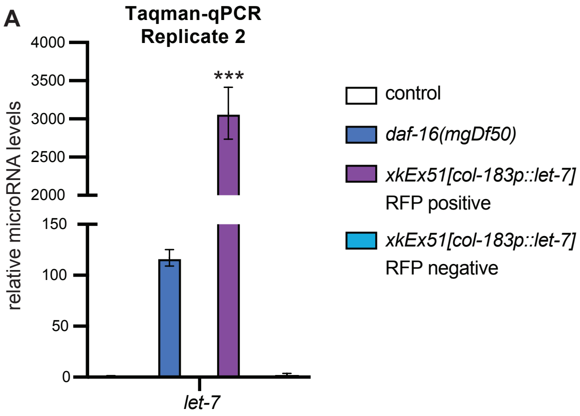

Fig. S1. Validation of overexpression of *let-7* during dauer. **A.** Taqman qPCR measuring *let-7* levels during dauer in the indicated genotypes with *daf-7(e1372); maIs105[col-19p::gfp]* in the background. Expression was normalized to U18 and then to the value in control *dauers*. Bars represent mean of two technical replicates and error bars represent standard deviation. P values were calculated using Student's t-test. *** $p<0.001$.

bioRxiv preprint doi: https://doi.org/10.64898/2026.07.18.739353; this version posted July 20, 2026. The copyright holder for this preprint (which was not certified by peer review) is the author/funder, who has granted bioRxiv a license to display the preprint in perpetuity. It is made available under a CC-BY 4.0 International license.

Figure S2

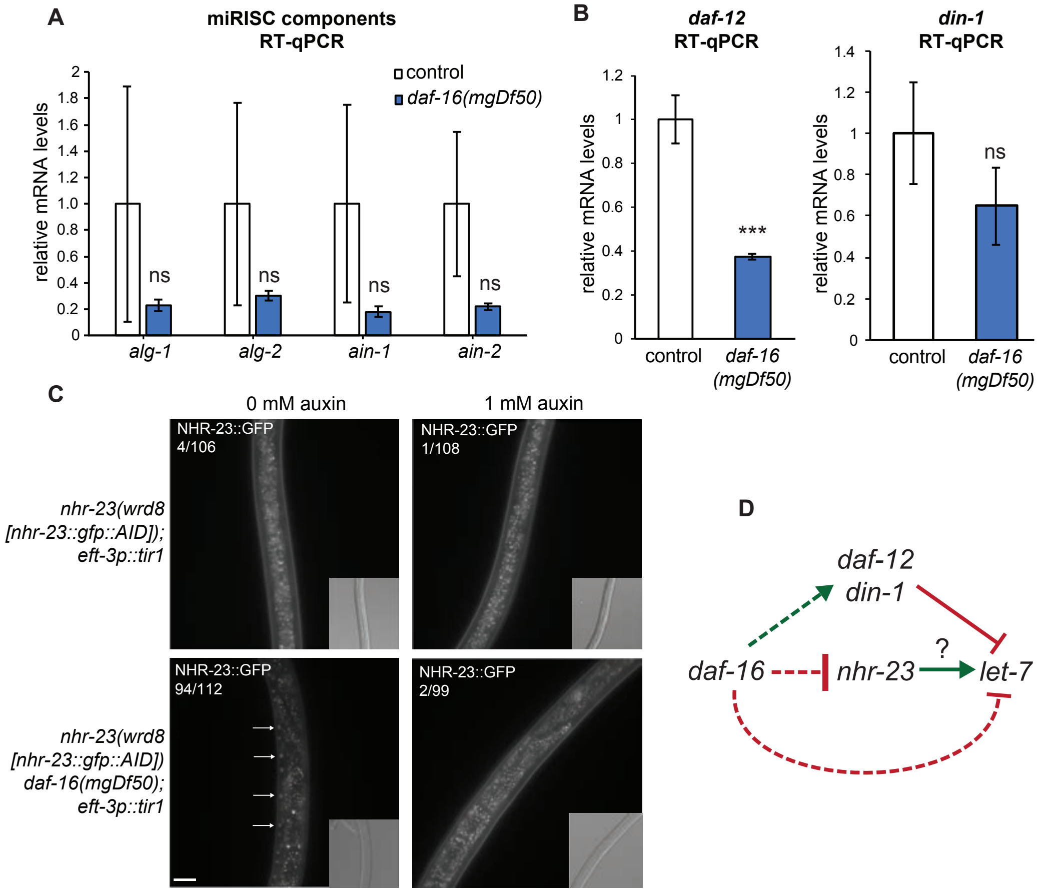

Fig. S2. *nhr-23* is upregulated and *daf-12* is downregulated in *daf-16(mgDf50); daf-7(e1372)* dauers. All strains in this figure have *daf-7(e1372)* in the background. **A, B.** RT-qPCR measuring expression of **A**. indicated components of the miRISC and **B.** *daf-12* and *din-1* in control and *daf-16(mgDf50)* dauers. Bars represent the mean of three biological replicates and error bars represent standard deviation. P values were calculated using Student's t-test. n.s., non significant.***p<0.001. **C.** Representative images showing NHR-23::GFP expression in the indicated genotypes and auxin conditions. Strains have *ieSi57[eft-3p::tir1].* The number of animals expressing NHR-23::GFP::AID is indicated. Arrows indicate seam cell nuclei. Images of 0 mM auxin condition on the left are reproduced from Figure 2E. Scale bar = 20μm **D.** Schematic summarizing the genetic interactions established in this figure and in Figure 2A–E. Dashed lines indicate possible involvement of additional factors. The question mark indicates the pathway investigated in Figure 2F–H.

bioRxiv preprint doi: https://doi.org/10.64898/2026.07.18.739353; this version posted July 20, 2026. The copyright holder for this preprint (which was not certified by peer review) is the author/funder, who has granted bioRxiv a license to display the preprint in perpetuity. It is made available under a CC-BY 4.0 International license.

Figure S3

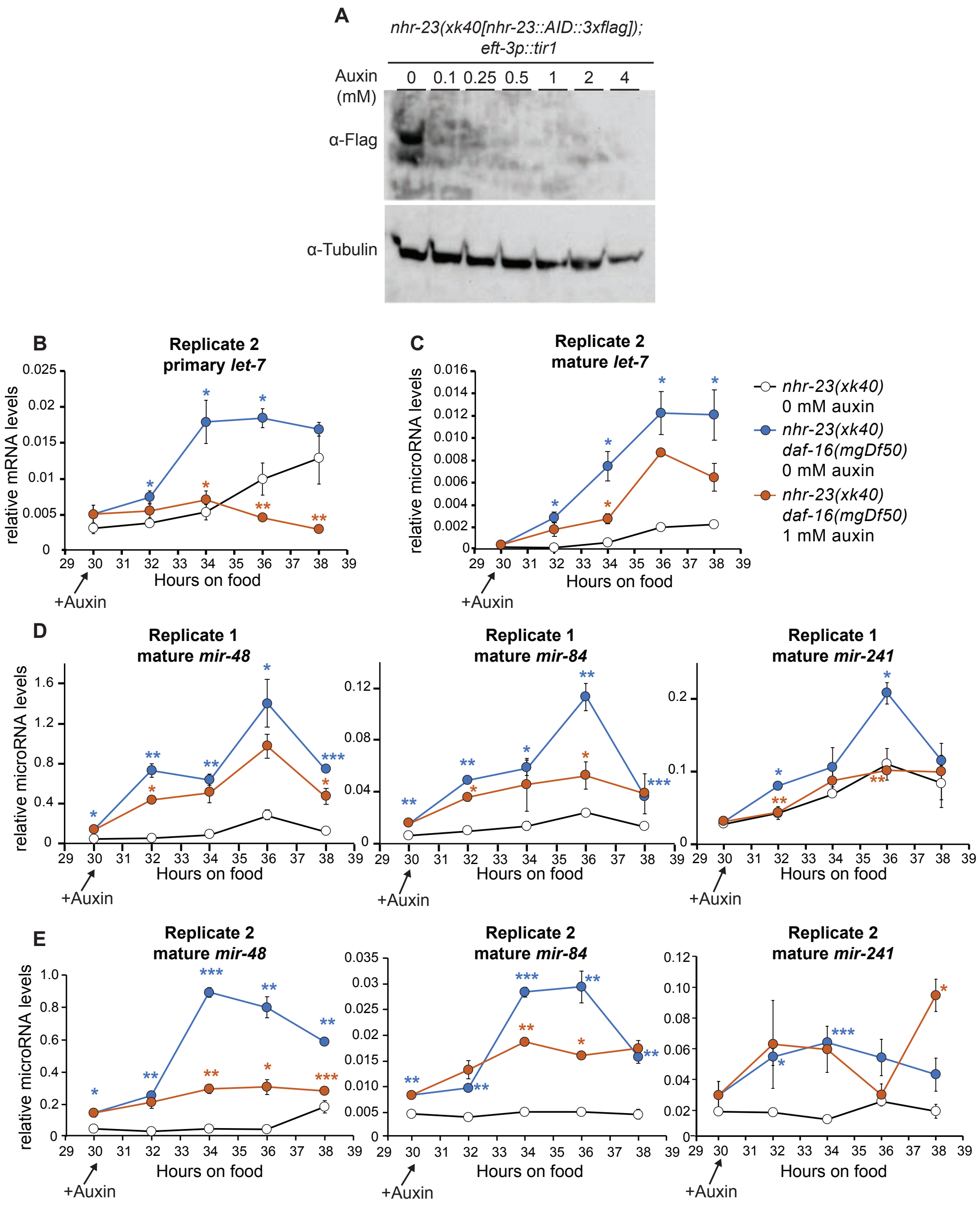

Fig. S3. NHR-23 mediates upregulation of the *let-7* family of microRNAs in *daf-16(mgDf50); daf-7(e1372)* dauers. A. Western blot showing NHR-23::AID::3xFlag levels in starved dauers after exposure to the indicated concentrations of auxin for 2 h. **B–E.** All strains have *daf-7(e1372); ieSi57[eft-3p::tir1]* in the background. RT-qPCR measuring primary *let-7* levels **(B)** and Taqman qPCR measuring mature *let-7* **(C)**, *mir-48, mir-84* and *mir-241* levels **(D-E)** in the indicated

bioRxiv preprint doi: https://doi.org/10.64898/2026.07.18.739353; this version posted July 20, 2026. The copyright holder for this preprint (which was not certified by peer review) is the author/funder, who has granted bioRxiv a license to display the preprint in perpetuity. It is made available under a CC-BY 4.0 International license.

genotypes. Worms were exposed to 0 mM or 1 mM auxin at 30 h. Each data point represents the mean of two technical replicates and error bars represent standard deviation. Blue asterisks indicate comparisons between *nhr-23::AID,* 0 mM and *nhr-23::AID daf-16(mgDf50),* 0 mM auxin. Orange asterisks indicate comparisons between *nhr-23::AID daf-16(mgDf50)* 0 mM auxin and *nhr-23::AID daf-16(mgDf50),* 1 mM auxin. P values were calculated using Student's t-test. *p<0.05, **p<0.01, ***p<0.001.

bioRxiv preprint doi: https://doi.org/10.64898/2026.07.18.739353; this version posted July 20, 2026. The copyright holder for this preprint (which was not certified by peer review) is the author/funder, who has granted bioRxiv a license to display the preprint in perpetuity. It is made available under a CC-BY 4.0 International license.

Figure S4

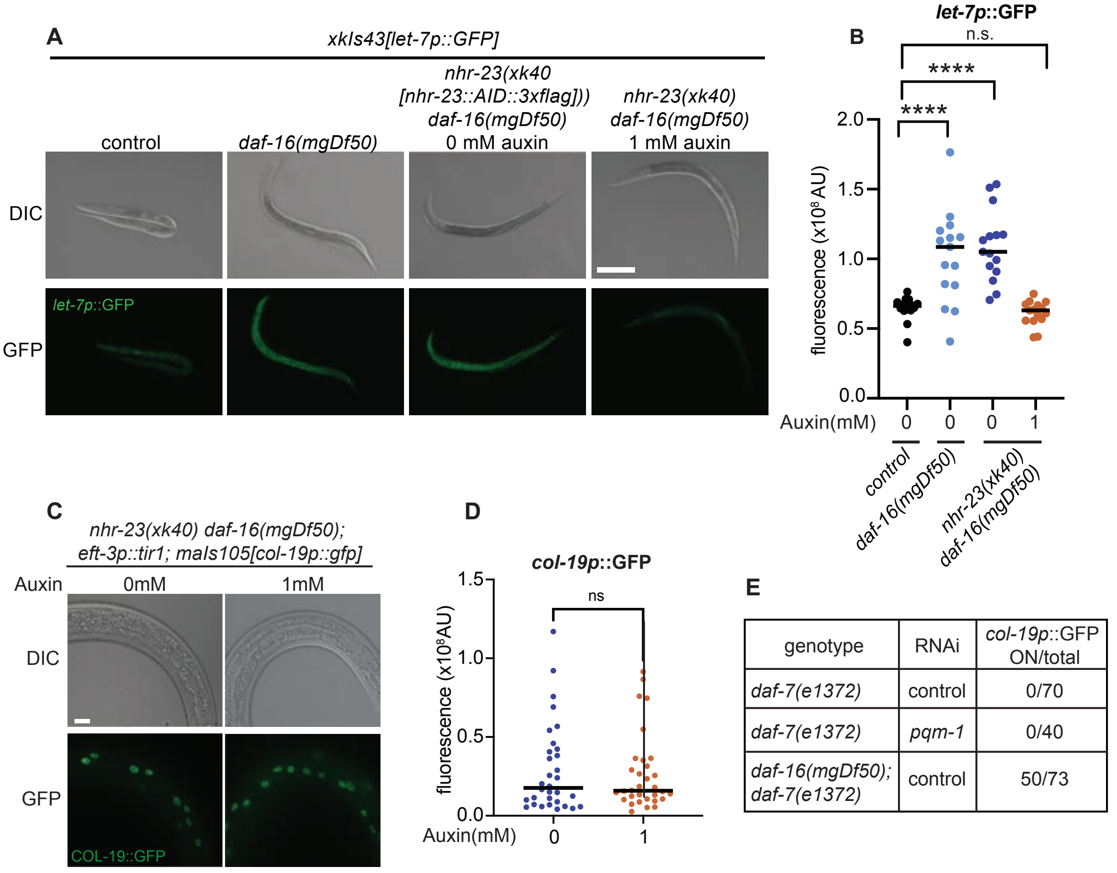

genotype	RNAi	*col-19p*::GFP ON/total
daf-7(e1372)	control	0/70
daf-7(e1372)	*pqm-1*	0/40
daf-16(mgDf50); daf-7(e1372)	control	50/73

Fig. S4. NHR-23 mediates upregulation of *let-7* transcription in *daf-16(mgDf50); daf-7(e1372)* during L2d. All strains have *daf-7(e1372).* **A.** Representative images of indicated genotypes at 38 h at 24°C. Animals were transferred to plates containing 0 mM or 1 mM auxin at 28 h. Scale bar = 100µm. **B.** Quantification of images shown in A. Each data point represents the mean fluorescence intensity from at least three hypodermal cells per animal. N = 14–20. P values were calculated using the Mann Whitney test. n.s., not significant; ****p<0.0001. **C.** Representative images of *col-19p*::GFP expression in dauers of indicated genotypes exposed to 0 mM or 1 mM auxin for 2 h. Scale bar = 10µm **D.** Quantification of images shown in C. Each data point represents the mean fluorescence intensity from at least three hypodermal cells per animal. N = 14–20. P values were calculated by using the Mann Whitney test. n.s., not significant; ****p<0.0001. **E.** Table showing number of dauers expressing *col-19p*::GFP in indicated conditions.

bioRxiv preprint doi: https://doi.org/10.64898/2026.07.18.739353; this version posted July 20, 2026. The copyright holder for this preprint (which was not certified by peer review) is the author/funder, who has granted bioRxiv a license to display the preprint in perpetuity. It is made available under a CC-BY 4.0 International license.

Figure S5

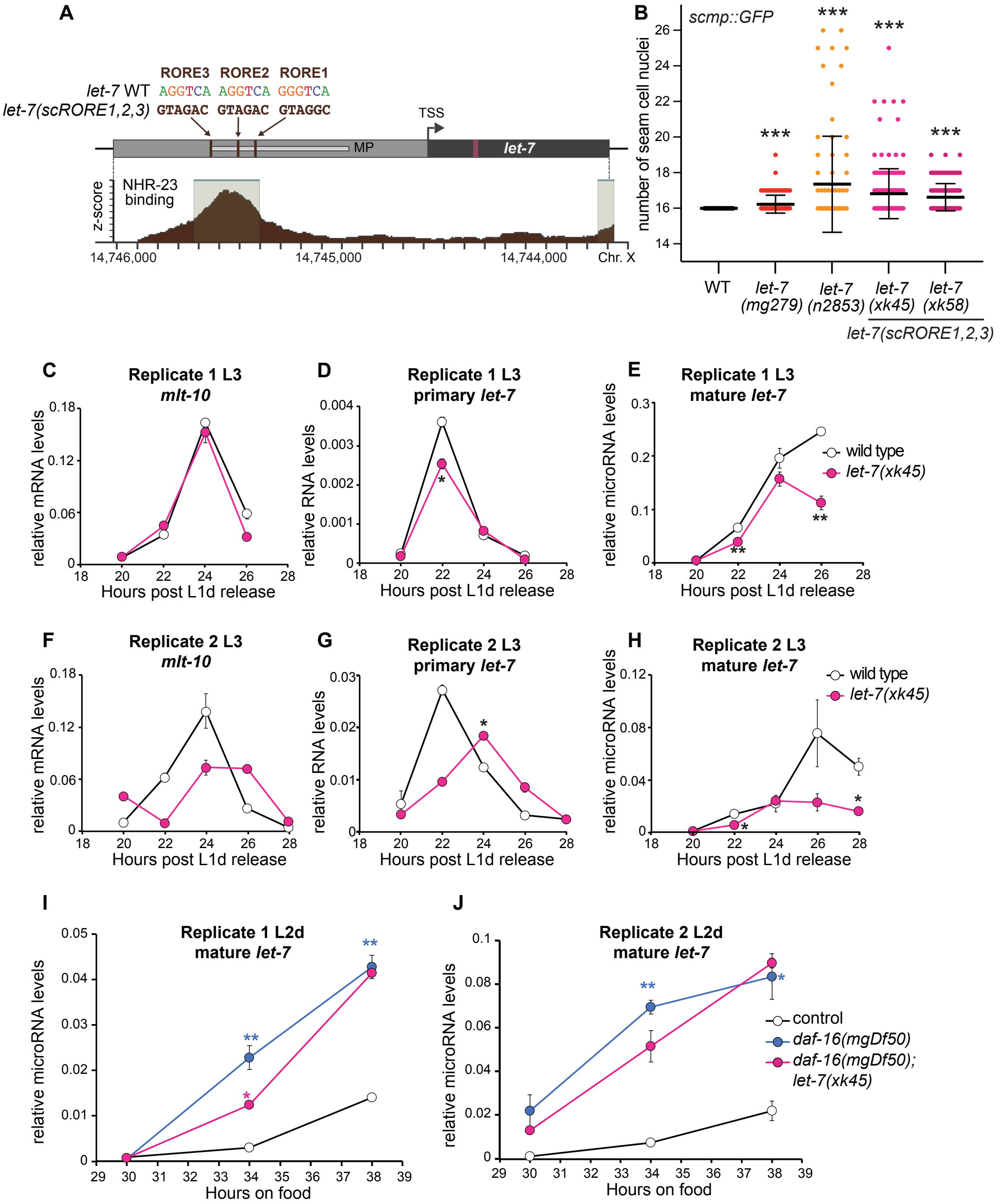

Fig. S5. ROREs mediate NHR-23 binding to the *let-7* promoter to promote transcription during continuous development and L2d. A. Schematic of the *let-7* promoter showing ROR binding element (RORE) sequences in wild-type and *let-7(scRORE1,2,3)* alleles. MP indicates the minimal promoter from Kai et al., 2013. TSS indicates the transcription start site. Adapted from

bioRxiv preprint doi: https://doi.org/10.64898/2026.07.18.739353; this version posted July 20, 2026. The copyright holder for this preprint (which was not certified by peer review) is the author/funder, who has granted bioRxiv a license to display the preprint in perpetuity. It is made available under a CC-BY 4.0 International license.

Patel et al., 2022. **B.** Number of seam cell nuclei in adults of the indicated genotypes. Each data point represents a single animal. Thick line indicates the mean of the distribution and error bars represent standard deviation. N = 67-169 for each genotype. P values were calculated using Student's t-test**.** ***p<0.001. **C–H.** Expression analysis during the L3 stage. RT-qPCR measuring *mlt-10* **(C,F)** and primary *let-7* **(D,G)** levels. **E, H.** Taqman qPCR measuring mature *let-7* expression during L3. **I, J**. Strains have *daf-7(e1372).* Taqman qPCR measuring mature *let-7* levels during L2d. Data points represent the mean of two technical replicates and error bars represent standard deviation. P values were calculated using Student's t-test. *p<0.05, **p<0.01. In G–H, blue asterisks indicate comparisons between control and *daf-16(mgDf50)*, while pink asterisks indicate comparisons between *daf-16(mgDf50)* and *daf-16(mgDf50); let-7(xk45(scRORE1,2,3))* animals. In D and G, comparisons were made between the amplitudes of the two gene expression curves.

bioRxiv preprint doi: https://doi.org/10.64898/2026.07.18.739353; this version posted July 20, 2026. The copyright holder for this preprint (which was not certified by peer review) is the author/funder, who has granted bioRxiv a license to display the preprint in perpetuity. It is made available under a CC-BY 4.0 International license.

Figure S6

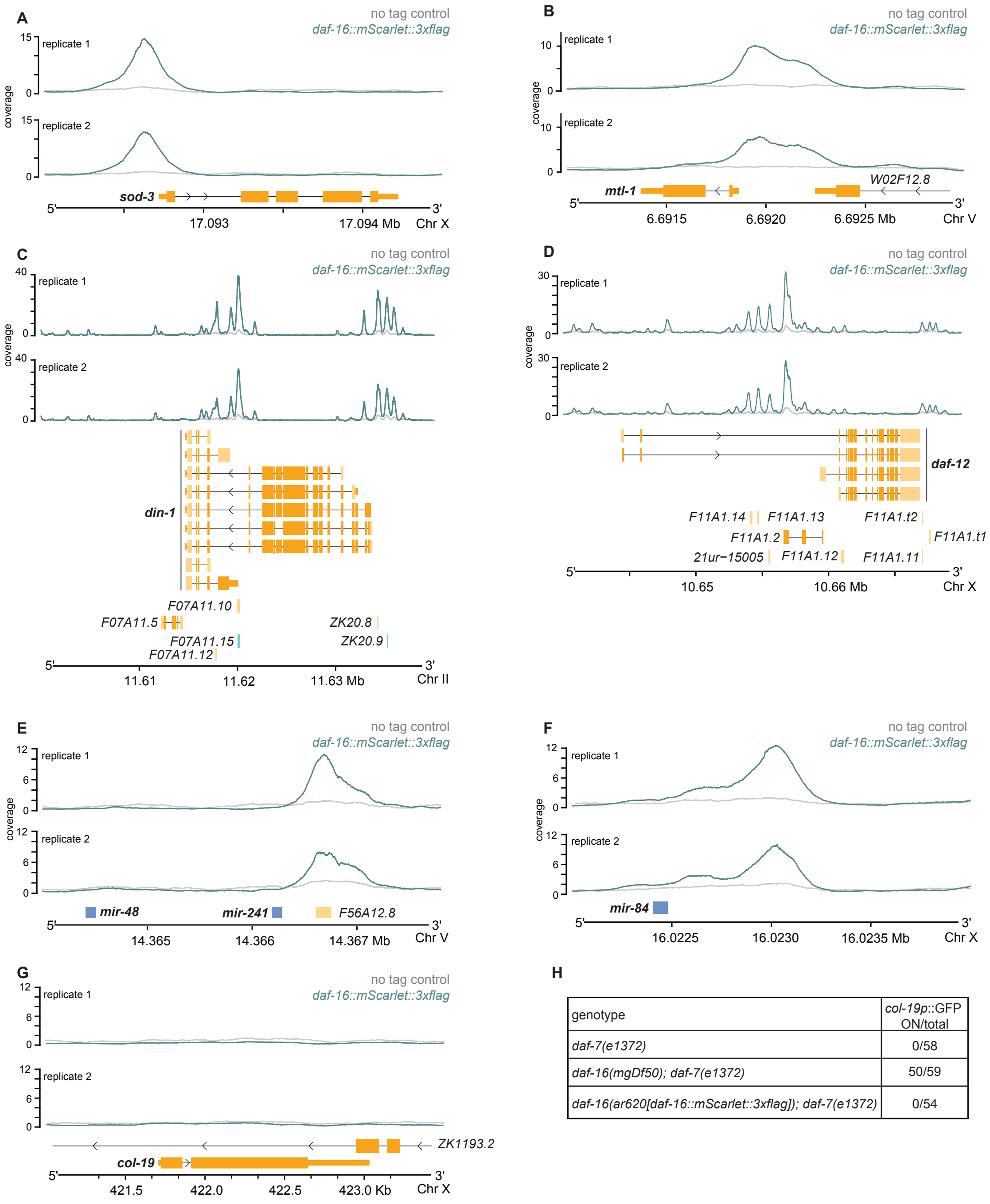

genotype	*col-19p*::GFP ON/total
daf-7(e1372)	0/58
daf-16(mgDf50); daf-7(e1372)	50/59
daf-16(ar620[daf-16::mScarlet::3xflag]); daf-7(e1372)	0/54

Fig. S6. DAF-16 binds upstream of several genes during dauer. ChIP-seq tracks for N2 and *daf-16::mScarlet::3xflag* dauers showing DAF-16 binding summits upstream of **A.** *sod-3,* **B.** *mtl-1*, **C.** *daf-12,* **D.***din-1,* **E.** *mir-241*, and **F.** *mir-84*. **G.** DAF-16 binding summits were not detected upstream of *col-19*. **H.** Strains contain *maIs105[col-19p::gfp]*. Number of dauers expressing *col-19p*::GFP is indicated.

bioRxiv preprint doi: https://doi.org/10.64898/2026.07.18.739353; this version posted July 20, 2026. The copyright holder for this preprint (which was not certified by peer review) is the author/funder, who has granted bioRxiv a license to display the preprint in perpetuity. It is made available under a CC-BY 4.0 International license.

Figure S7

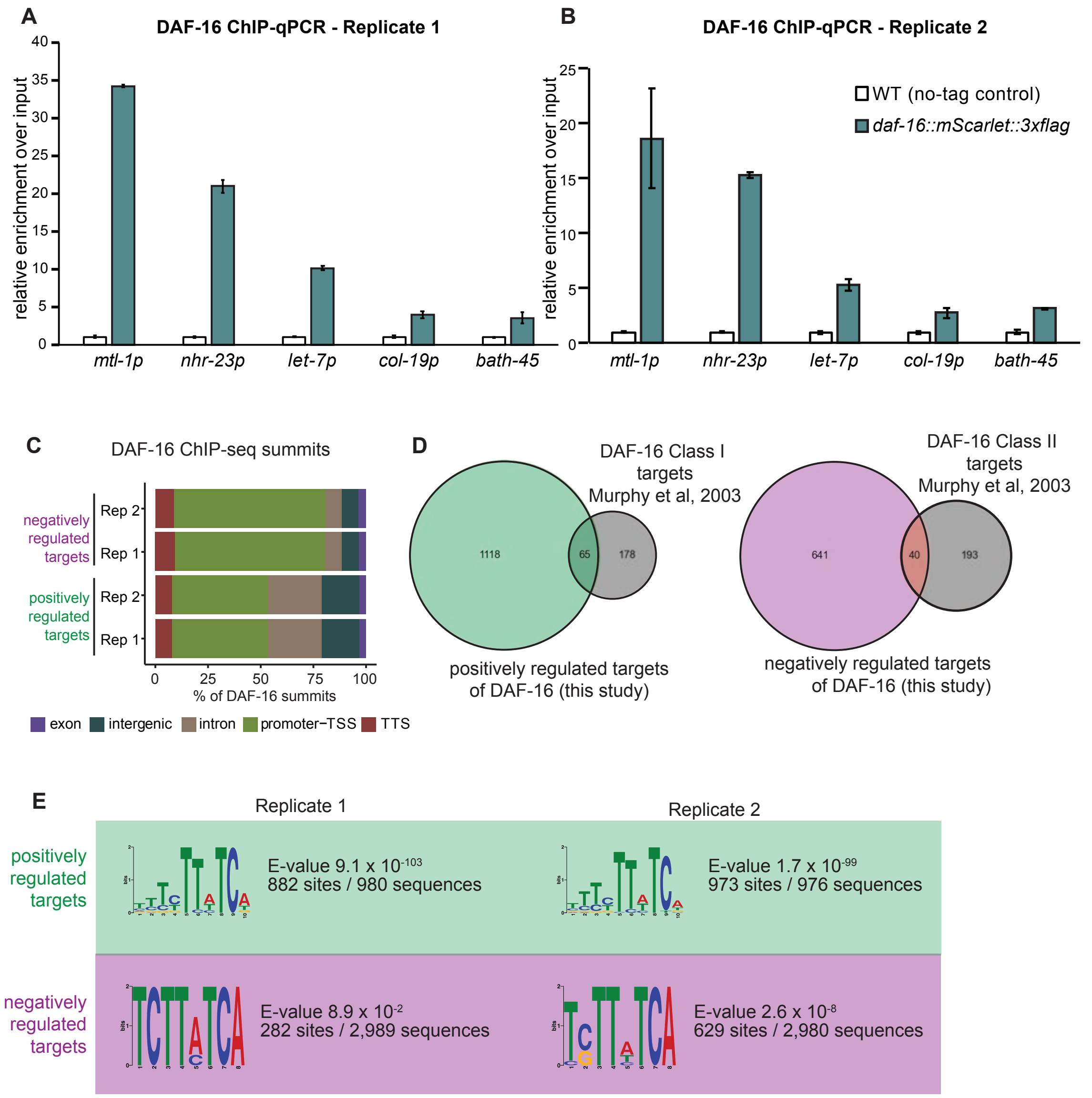

Fig. S7. Analysis of DAF-16 ChIP-seq data. A, B. ChIP-qPCR validation of DAF-16 enrichment at promoter region of *nhr-23 and let-7* during dauer. Two biological replicates are shown. Bars represent the mean of two technical replicates from one biological replicate and error bars represent standard deviation. The known DAF-16 target *mtl-1* and a heterochromatinized gene *bath-45* were used as positive and negative controls, respectively. **C.** Distribution of DAF-16 consensus binding summits across annotated genomic regions. **D.** Overlap between DAF-16

bioRxiv preprint doi: https://doi.org/10.64898/2026.07.18.739353; this version posted July 20, 2026. The copyright holder for this preprint (which was not certified by peer review) is the author/funder, who has granted bioRxiv a license to display the preprint in perpetuity. It is made available under a CC-BY 4.0 International license.

targets identified in this study and targets previously reported by Murphy et al., 2003. **E.** MEME-ChIP analysis of positively and negatively regulated DAF-16 targets.

bioRxiv preprint doi: https://doi.org/10.64898/2026.07.18.739353; this version posted July 20, 2026. The copyright holder for this preprint (which was not certified by peer review) is the author/funder, who has granted bioRxiv a license to display the preprint in perpetuity. It is made available under a CC-BY 4.0 International license.

Figure S8

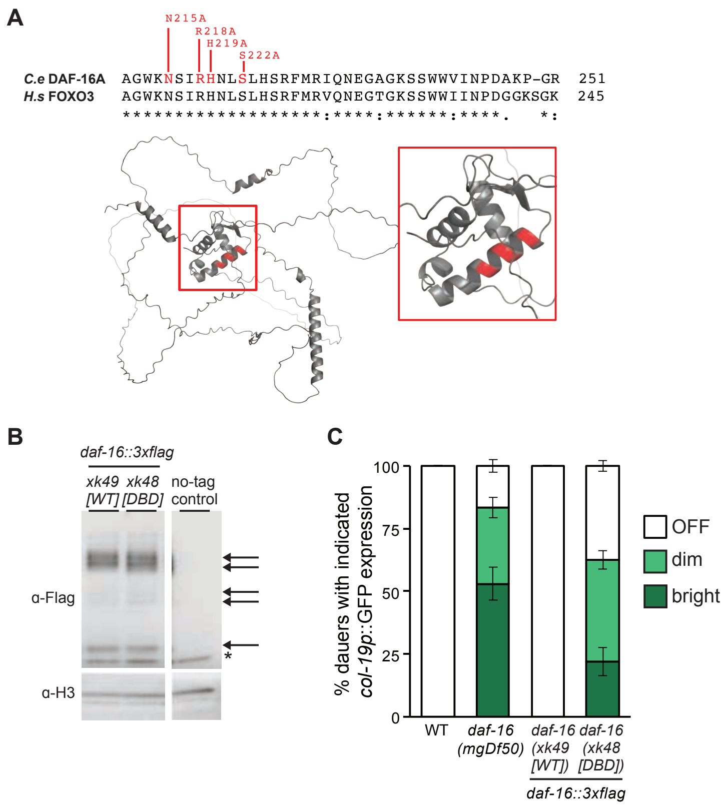

Fig. S8. *daf-16::3xflag(DBD)* is a reduced function allele of *daf-16*. A. Top: Alignment of the FOXO domain of *C. elegans* DAF-16A and human FOXO3. Labeled residues are required for DNA binding of human FOXO3 (Tsai et al., 2007). Bottom: Predicted structure of *C. elegans* DAF-16A generated using AlphaFold. Conserved residues mutated to alanine in the DNA-binding defective allele *daf-16*(*xk48[daf-16::3xflag(DBD)])* are highlighted in red. **B.** Western blot of DAF-16::3xFLAG in *daf-7(e1372); daf-16*(*xk49[daf-16::3xflag(WT)])* and *daf-7(e1372); daf-16*(*xk48[daf-16::3xflag(DBD)])* dauers. Arrows indicate bands corresponding to FLAG-tagged DAF-16 isoforms. Asterisk indicates a non-specific band. **C.** Percentage of *daf-7(e1372)* dauers exhibiting *col-19p*::GFP expression in the indicated genotypes at 52 h at 25°C. N = 200–300 animals per genotype.

bioRxiv preprint doi: https://doi.org/10.64898/2026.07.18.739353; this version posted July 20, 2026. The copyright holder for this preprint (which was not certified by peer review) is the author/funder, who has granted bioRxiv a license to display the preprint in perpetuity. It is made available under a CC-BY 4.0 International license.

Figure S9

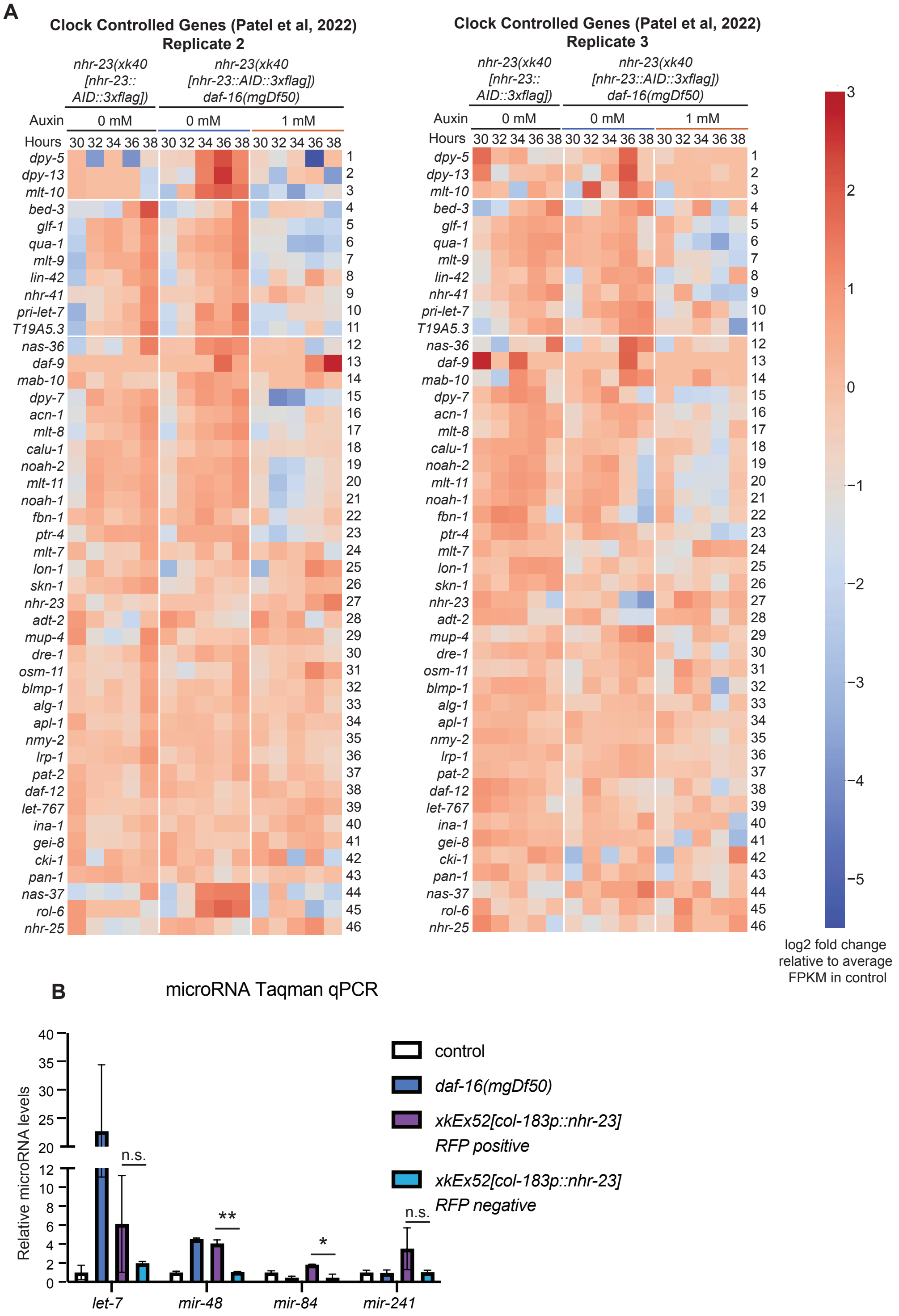

Fig. S9. Suppression of *nhr-23* by DAF-16 affects expression of genes associated with continuous development. A. Heat maps showing expression profiles of Clock Controlled Genes (CCGs) in the indicated genotypes during development L2d development. Strains have *daf-*

bioRxiv preprint doi: https://doi.org/10.64898/2026.07.18.739353; this version posted July 20, 2026. The copyright holder for this preprint (which was not certified by peer review) is the author/funder, who has granted bioRxiv a license to display the preprint in perpetuity. It is made available under a CC-BY 4.0 International license.

7(e1372); ieSi57. Animals were transferred to plates containing 0 mM or 1 mM auxin at 30 h and maintained at 24°C. **B.** Taqman qPCR measuring mature *let-7, mir-48, mir-84* and *mir-241* levels in the indicated genotypes during dauer. Strains have *daf-7(e1372); maIs105[col-19p::gfp]*. P values were calculated using Student's t test. *p<0.05, **p<0.01; n.s., not significant.

bioRxiv preprint doi: https://doi.org/10.64898/2026.07.18.739353; this version posted July 20, 2026. The copyright holder for this preprint (which was not certified by peer review) is the author/funder, who has granted bioRxiv a license to display the preprint in perpetuity. It is made available under a CC-BY 4.0 International license.

Figure S10

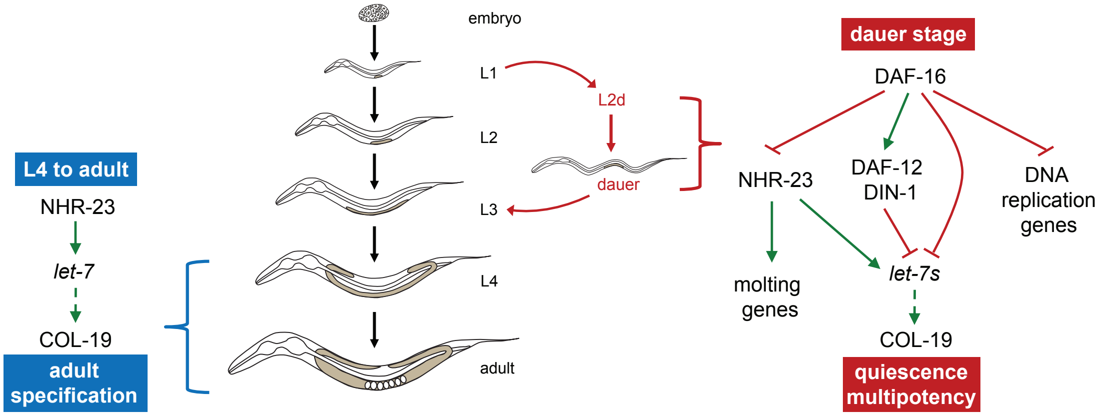

Fig. S10. Model: DAF-16 modulates NHR-23 and other pro-growth genes to establish quiescence during dauer. DAF-16 binds upstream of *nhr-23* and suppresses its expression. Because NHR-23 is an essential transcription factor for molting genes (Kouns et al., 2011) and for *let-7* transcription (Patel, Galagali et al., 2022), DAF-16-mediated repression of *nhr-23* may contribute to induction of developmental quiescence. DAF-16 may also promote expression of *daf-12* and *din-1*, which together inhibit *let-7* transcription during dauer (Hammell et al., 2009). In addition, DAF-16 may directly repress transcription of *let-7* and other pro-growth genes, thereby establishing and reinforcing quiescence during dauer.

bioRxiv preprint doi: https://doi.org/10.64898/2026.07.18.739353; this version posted July 20, 2026. The copyright holder for this preprint (which was not certified by peer review) is the author/funder, who has granted bioRxiv a license to display the preprint in perpetuity. It is made available under a CC-BY 4.0 International license.

Tables

Table S1. Transcription factors regulated by DAF-16

Gene stable ID	Gene name	Type of target
WBGene00000431	*ceh-6*	positively regulated
WBGene00000459	*ceh-38*	positively regulated
WBGene00000908	*daf-12*	positively regulated
WBGene00000910	*daf-14*	positively regulated
WBGene00000912	*daf-16*	positively regulated
WBGene00001182	*egl-13*	positively regulated
WBGene00001194	*egl-27*	positively regulated
WBGene00001249	*elt-1*	positively regulated
WBGene00001439	*fkh-7*	positively regulated
WBGene00001707	*grh-1*	positively regulated
WBGene00001837	*hda-4*	positively regulated
WBGene00001957	*hlh-13*	positively regulated
WBGene00002261	*ldb-1*	positively regulated
WBGene00003163	*mdl-1*	positively regulated
WBGene00003167	*mec-3*	positively regulated
WBGene00003609	*nhr-10*	positively regulated
WBGene00003647	*nhr-57*	positively regulated
WBGene00003659	*nhr-69*	positively regulated
WBGene00003687	*nhr-97*	positively regulated
WBGene00003933	*pat-9*	positively regulated
WBGene00004013	*pha-4*	positively regulated
WBGene00004024	*php-3*	positively regulated
WBGene00004771	*sem-2*	positively regulated
WBGene00004773	*sem-4*	positively regulated
WBGene00004804	*skn-1*	positively regulated
WBGene00006462	*svh-5*	positively regulated
WBGene00006543	*tbx-2*	positively regulated
WBGene00006959	*xbp-1*	positively regulated
WBGene00006970	*zag-1*	positively regulated
WBGene00008640	F10B5.3	positively regulated
WBGene00008748	*yap-1*	positively regulated
WBGene00009224	F28F8.7	positively regulated
WBGene00011259	*miz-1*	positively regulated
WBGene00013970	*klu-1*	positively regulated
WBGene00016091	*nhr-30*	positively regulated

bioRxiv preprint doi: https://doi.org/10.64898/2026.07.18.739353; this version posted July 20, 2026. The copyright holder for this preprint (which was not certified by peer review) is the author/funder, who has granted bioRxiv a license to display the preprint in perpetuity. It is made available under a CC-BY 4.0 International license.

WBGene00016937	*tag-294*	positively regulated
WBGene00016997	*cebp-1*	positively regulated
WBGene00017430	*bcl-11*	positively regulated
WBGene00020251	*camt-1*	positively regulated
WBGene00020368	*ast-1*	positively regulated
WBGene00021610	*nhr-237*	positively regulated
WBGene00021931	*znf-782*	positively regulated
WBGene00022861	*dve-1*	positively regulated
WBGene00001977	*hmg-12*	negatively regulated
WBGene00002324	*let-49*	negatively regulated
WBGene00003107	*mab-10*	negatively regulated
WBGene00003607	*nhr-8*	negatively regulated
WBGene00003622	*nhr-23*	negatively regulated
WBGene00003626	*nhr-32*	negatively regulated
WBGene00003645	*nhr-55*	negatively regulated
WBGene00003670	*nhr-80*	negatively regulated
WBGene00003681	*nhr-91*	negatively regulated
WBGene00003689	*nhr-99*	negatively regulated
WBGene00003691	*nhr-101*	negatively regulated
WBGene00004078	*pos-1*	negatively regulated
WBGene00009937	*lsl-1*	negatively regulated
WBGene00010545	*cbp-2*	negatively regulated
WBGene00017687	*ets-4*	negatively regulated
WBGene00017690	*ceh-60*	negatively regulated

Table S2. List of strains used in this study

Strain name	Genotype	Used in figures	Source/Reference
VT1777	*daf-7(e1372) III; maIs105[col-19p::gfp] V*	1B, 1F, 1G, 2D, S1A, S2A, S2B, S4E, S6H	e1372: Riddle et al, Nature, 1981<br>maIs105: Feinbaum, Ambros, Dev Biol, 1999
XV36	*daf-16(mgDf50) I; daf-7(e1372) III; maIs105[col-19p::gfp] V*	1B, 1C, 1D, 2D, S2A, S2B, S4E, S6H	mgDf50: Ogg et al, Nature, 1997
XV226	*daf-16(mgDf50)I; daf-7(e1372) III; maIs105[col-19p::gfp] V; let-7(n2853) X*	1C, 1D	n2853: Reinhart et al, Nature, 2000
XV162	*daf-16(mgDf50); daf-7(e1372) III; nDf51 maIs105[col-19p::gfp] V*	1C, 1D	nDf51: Abbott et al, Dev Cell, 2005
QK244	*daf-7(e1372) III; maIs105[col-19p::gfp] V; xkEx51[col-183p::let-7::let-7 3'UTR + myo-2p::mCherry::unc-54 3'UTR]*	1F, 1G, S1A	xkEx51: this study. Look at Methods section for details
QK152	*daf-7(e1372) III; xkIs43[let-7p::gfp]*	2A, 2B, S4A, S4B	xkIs43: this study. Look at Methods section for details
XV285	*daf-16(mgDf50) I; daf-7(e1372) III; xkIs43[let-7p::gfp]*	2A, 2B, S4A, S4B	
QK221	*nhr-23(wrd8[nhr-23::gfp::aid*::3xflag]) I; ieSi57[eft-3p::tir1::mRuby::unc-54 3'UTR + Cbr-unc-119(+)] II; daf-7(e1372) III*	2E, S2C	wrd8: Ragle JM, et al. Development. 2020<br>ieSi57: Zhang L, et al. Development. 2015
QK222	*nhr-23(wrd8[nhr-23::gfp::aid*::3xflag]) daf-16(mgDf50) I; ieSi57[eft-3p::tir1::mRuby::unc-54 3'UTR + Cbr-unc-119(+)] II; daf-7(e1372) III*	2E, S2C	
QK216	*nhr-23(xk40[nhr-23::aid::3xflag]) I; ieSi57[eft-3p::tir1::mRuby::unc-54 3'UTR + Cbr-unc-119(+)] II; daf-7(e1372) III; maIs105[col-19p::gfp] V*	2F, 4A, S3B, S3C, S3D, S3E, S4C, S4D	xk40: this study. Look at Methods section for details
QK215	*daf-16(mgDf50) nhr-23(xk40[nhr-23::aid::3xflag]) I; ieSi57[eft-3p::tir1::mRuby::unc-54 3'UTR + Cbr-unc-119(+)] II; daf-7(e1372) III; maIs105[col-19p::gfp] V*	2F, 4A, S3B, S3C, S3D, S3E, S4C, S4D	
QK218	*daf-7(e1372) III; maIs105[col-19p::gfp] V; let-7(xk45[scRORE1,2,3]) X*	2G, 2H, S5I, S5J	xk45: this study. Look at Methods section for details
QK219	*daf-16(mgDf50) I; daf-7(e1372) III; maIs105[col-19p::gfp] V; let-7(xk45[scRORE1,2,3]) X*	2G, 2H, S5I, S5J	
GS8924	*daf-16(ar620[daf-16::zf1-wrmScarlet-3xflag]) I*	3A, 3B, 3C, 3D, 3E, S6A	Gift from Iva Greenwald
QK230	*nhr-23(wrd33[nhr-23::30aa linker:mScarlet:SEC:3XMyc]) I; daf-7(e1372) III*	3F	wrd33: Myles KM, et al. MicroPubl Biol 2023
QK231	*nhr-23(wrd33[nhr-23::30aa linker:mScarlet:SEC:3XMyc]) daf-16(mgDf50) I; daf-7(e1372) III*	3F	

QK232	*daf-16(xk48[daf-16::3xflag(DBD/N215A R218A H219A S222A)]) nhr-23(wrd33[nhr-23::30aa linker:mScarlet:SEC:3XMyc]) I; daf-7(e1372) III*	3F	xk48: this study. Look at Methods section for details
QK245	*daf-7(e1372) III; maIs105[col-19::gfp] V; xkEx52[col-183p::nhr-23::3xflag::nhr-23 3'UTR + myo-2p::mCherry::unc-54 3'UTR]*	4B, S9B	xkEx52: this study. Look at Methods section for details
QK200	*nhr-23(xk40[nhr-23::aid::3xflag]) I; ieSi57[eft-3p::tir1::mRuby::unc-54 3'UTR + Cbr-unc-119(+)] II*	S3A	
XV288	*nhr-23(xk40[nhr-23::aid::3xflag]) daf-16(mgDf50) I; ieSi57[eft-3p::tir1::mRuby::unc-54 3'UTR + Cbr-unc-119(+)] II; daf-7(e1372) III; xkIs43[let-7p::gfp]*	S4A, S4B	
QK206	*let-7(mg279) X; wIs54[scm::GFP]*	S5B	mg279: Reinhart et al, Nature, 2000
QK036	*let-7(n2853) X; wIs54[scm::GFP]*	S5B	
QK246	*let-7(xk45[scRORE-1,2,3]) X; wIs54 line 1*	S5B	xk45: this study. Look at Methods section for details
QK247	*let-7(xk58[scRORE-1,2,3]) X; wIs54 line 2*	S5B	xk58: this study. Look at Methods section for details
JR672	*wIs54[scm::GFP]*	S5B	
QK350	*let-7(xk45[scRORE-1,2,3]) X*	S5C, S5D, S5E, S5F, S5G, S5H	
XV229	*daf-16(ar620[daf-16::mScarlet]) I; daf-7(e1372) III; maIs105 V*	S6H	
QK233	*daf-16(xk48 [daf-16::3xflag(DBD/N215A R218A H219A S222A)]); daf-7(e1372) III; maIs105[col-19p::gfp]*	S8B, S8C	
QK234	*daf-16(xk49 [daf-16::3xflag]); daf-7(e1372) III; maIs105[col-19p::gfp]*	S8B, S8C	

Table S3. Oligonucleotides used in this study.

Oligo ID	Sequence	Notes
oHG318	AATCGCAAACCTTCAACGAAGAG	primers to clone *col-183p* from the *C. elegans* genome
oHG319	GGTTGACTGGTTGCTGTTGCT	
oHG320	AAATTTAAAGGCCGGCCAATCGCAAACCTTCAACGAAGAG	primers to clone *col-183p* into the overexpression construct with FseI and XmaI sites
oHG321	CCCGGGGGCGCGCCGGTTGACTGGTTGCTGTTGCT	
oHG345	ATTCTAGATGAGTAGCCCACCTA	primers to clone primary *let-7*(C05G5.6.2) from the *C. elegans* genome
oHG346	TGAAAACTAAAAACACTAACAAAGAA	
oHG336	CGCGCCCCCGGGATTCTAGATGAGTAG	primers to clone primary *let-7*(C05G5.6.2) into the overexpression construct with XmaI and AsiSI sites
oHG337	GCCCCTAGGCGATCGCTGAAAACTAAAAACACTAAC	
oHG348	TTTATTTGGGGATAGGTACCG	primers to clone *nhr-23::3xflag* from the *C. elegans* genome QK159
oHG349	ATGCAAGCTGTAGAGCAGAAGT	
oHG327	GACGAAAGGGCCCCTAGGCGATCGCTTTATTTGGGGATAGG	primers to clone *nhr-23::3xflag* into the overexpression construct with XmaI and ApaI sites
oHG328	CGCGCCCCCGGGATGCAAGCTGTAGAG	
OHG95	TATTCCCCTCCACCATCCCAT	qPCR primers for *nhr-23*
OHG96	TGGAGTCGTAGGAGTCGCAG	
OHG97	CTACGGAAAGGAAAGCCCGT	qPCR primers for *mlt-10*
OHG98	GGAACTTTTCGGCTTCAGCG	
oTH1269	ACGCTCGTGATGAGTTCAAG	qPCR primers for *eft-2*
oTH1270	ATTTGGTCCAGTTCCGTCTG	
oTH1319	GATCCTCCGATGAACGAAAA	qPCR primers for *daf-12*
oTH1320	CTCTTCGGCTTCACCAGAAC	
oHG467	ATCCACGTCGAGACTCTGTG	qPCR primers for *din-1*
oHG468	CGACGAAAGTGCAAGACAGC	
oHG193	CAAGCAGGCGATTGGTGGA	qPCR primers for *pri-let-7*
oHG194	GACGCAGCTTCGAAGAGTTCTGTC	

oTH1	GAGTGCACTGTCGCCAAGTATTTCT	qPCR primers for *alg-1*
oTH2	GCAGAACGAGCTGTAGCCTTAATCA	
oTH3	GGAAAATGATCTCAGGATGTTCACG	qPCR primers for *alg-2*
oTH4	ACTTCGGCATAGATCGGAGTCTTTC	
oVK120	AAGGGAGAGGACAATCAACG	qPCR primers for *ain-1*
oVK121	CTTGGACGGATTGTTTGGATCAT	
oVK60	ATAAGGATCAAAAGAACGATCACG	qPCR primers for *ain-2*
oVK61	TGTGTCACCTCGATCTTTGCA	
oHG231	TGAGCACTCTAATCCTTTGCAC	ChIP-qPCR primers for *mtl-1* promoter
oHG232	ACGTGAATGTTGCAAACACCT	
oHG235	TCCATCTCTCTTGGAAACACAT	ChIP-qPCR primers for *col-19* promoter
oHG236	ACACCTTCAAACCTAACCAGTGT	
oHG251	CACCACCATCATCGCAAACC	ChIP-qPCR primers for *nhr-23* promoter
oHG252	AACGGTACGGTATGCCTCC	
oHG255	GTCCTCCATGGTATCAGGCAA	ChIP-qPCR primers for *let-7* promoter
oHG256	GCGTCGTTTGCGTATGTGTA	
oJK131	ATTTAATTTGTTTTCAGAAGGCAGC	ChIP-qPCR primers for *bath-45*, coding sequence that is heterochromatinized during continuous development
oJK132	GTGCTTTCTGCATCAGAGCC	
oHG308	GATCGGCCTGGAGGCTCAGG	crRNA to generate *nhr-23(xk40)*, injected into QK159
oHG309	TCATGGTCCTTGTAATCTCCGTCGTGATCTTTATAGTCCGATCCCGATCCCTTCACGAACGCCGCCGCCTCCGGGCCACCGCTTGATTTTTGGCAGGAAACCATCACGTTCTTCCGGTATGATCTCACCGGTGGCCATCCCACAACTTGTGCCTTGGCCGGAGGTTTGGCTGGATCTTTAGGCATTCCTGAGCCTCCTCCTGATCCTCCTGGACGGTCTGCAGTGAATAGCTCTTTGTAGAGGGCAGGAAGCTTTTCAGATGATGT	Repair template to generate *nhr-23(xk40)*, injected into QK159
oHG282	CGCAGTGCTAGCCGTTGCAC	crRNA to generate *let-7(xk45)*, injected into QK198

oHG368	AAACAAGTCTACGTGCAACGGCTAGCACTGCGTGACACCCGATTAAATTCGCGTAGATGTTTTTTTTTTCTCTCTCTCTCTTTCGAATCGGATAAAAAAGAAATGGAACTTTTGTTCCACTTTTGATGGTATTTTATTGCGGCTTGCAGGATTTTGCAACATGTGCATTCGAGGGTAAAGGAAGAAGG	Repair template to generate *let-7(xk45),* injected into QK198
oAA1785	CTCTCTTTCGAACAACACCA	crRNA to generate *daf-16(xk49)*
oAA1786	ATGAGCTGAGTCAAGCTGGA	
oAA1789	GGATGTTGATGCATTGATCAGACATGAGCTGAGTCAAGCTGGAGGACAGCATATTCATTTTGATTTGGGATCGGACTACAAAGACCATGACGGTGATTATAAAGATCATGATATCGATTACAAGGATGACGATGACAAGTAAATTCTCTTCATTTTGTTTCCGCTGGTGTTGTTCGAAAGAGAGATAGCAAAGCAGCGAG	repair template to generate *daf-16(xk49)*
oAA1817	AATGAAGAGACAGATTGTGA	crRNA to generate *daf-16(xk48)*, round 1
oAA1818	GGCAATTTCCAAATAAAAATTATCATAAAATCTTGCAGGCCTCGATCGCCGCTAATCTGGCTCTTCATTCTCGTTTCATGCGAATTCAGAATGAAGG	Repair template to generate *daf-16(xk48),* round 1
oAA1926	AGAATGAAGAGACAGATTAG	crRNAs to generate *daf-16(xk48)*, round 2, injected into *daf-16::flag (N215A R218A H219A)*
oAA1927	TTCATGCGAATTCAGAATGA	
oAA1928	ccaaataaaaattatcataaaatcttgcaggcCTCGATCgctgctAATCTGgCTCTTCATTCTCGTTTtATGaGAATcCAaAAcGAgGGAGCCGGAAAGAGCTCGTGGTGGGTTATTAATCC	Repair template to generate *daf-16(xk48),* round 2, injected into *daf-16::flag (N215A R218A H219A)*

bioRxiv preprint doi: https://doi.org/10.64898/2026.07.18.739353; this version posted July 20, 2026. The copyright holder for this preprint (which was not certified by peer review) is the author/funder, who has granted bioRxiv a license to display the preprint in perpetuity. It is made available under a CC-BY 4.0 International license.

Dataset S1 (separate file). Positively regulated targets of DAF-16 during dauer.

Dataset S2 (separate file). Negatively regulated targets of DAF-16 during dauer.

Dataset S3 (separate file). Gene Ontology analysis of the positively regulated targets of DAF-16.

Dataset S4 (separate file). Gene Ontology analysis of the negatively regulated targets of DAF-16.

Dataset S5 (separate file). RNA-seq analysis of Clock Controlled Genes and *nhr-23* targets.

References

Abbott, A.L., Alvarez-Saavedra, E., Miska, E.A., Lau, N.C., Bartel, D.P., Horvitz, H.R., and Ambros, V. (2005). The *let-7* microRNA family members mir-48, mir-84, and mir-241 function together to regulate developmental timing in *Caenorhabditis elegans*. *Dev Cell 9*, 403–414.

Feinbaum, R., Ambros, V. (1999), The timing of *lin-4* RNA accumulation controls the timing of postembryonic developmental events in *Caenorhabditis elegans*, *Dev Biol* 210(1), 87–95

Hammell, C.M., Karp, X., and Ambros, V. (2009). A feedback circuit involving *let-7*-family miRNAs and DAF-12 integrates environmental signals and developmental timing in *Caenorhabditis elegans*. *Proc Natl Acad Sci USA 106*, 18668–18673.

Kai, Z.S., Finnegan, E.F., Huang, S., and Pasquinelli, A.E. (2013). Multiple cis-elements and trans-acting factors regulate dynamic spatio-temporal transcription of *let-7* in *Caenorhabditis elegans*. *Dev Biol 374*, 223–233.

Kouns, N.A., Nakielna, J., Behensky, F., Krause, M.W., Kostrouch, Z., and Kostrouchova, M. (2011). NHR-23 dependent collagen and hedgehog-related genes required for molting. *Biochem Biophys Res Commun 413*, 515–520.

Murphy, C.T., McCarroll, S.A., Bargmann, C.I., Fraser, A., Kamath, R.S., Ahringer, J., Li, H., and Kenyon, C. (2003). Genes that act downstream of DAF-16 to influence the lifespan of *Caenorhabditis elegans*. *Nature 424*, 277–283.

bioRxiv preprint doi: https://doi.org/10.64898/2026.07.18.739353; this version posted July 20, 2026. The copyright holder for this preprint (which was not certified by peer review) is the author/funder, who has granted bioRxiv a license to display the preprint in perpetuity. It is made available under a CC-BY 4.0 International license.

Myles, K.M., Ragle, J.M., Ward, J.D. (2023). An *nhr-23::mScarlet::3xMyc* knock-in allele for studying spermatogenesis and molting. *microPublication Biology*.

Ogg, S., Paradis, S., Gottlieb, S., Patterson, G.I., Lee, L., Tissenbaum, H.A., Ruvkun, G. (1997). The Fork head transcription factor DAF-16 transduces insulin-like metabolic and longevity signals in *C. elegans*. *Nature* 389(6654), 994–999.

Patel, R., Galagali, H., Kim, J.K., and Frand, A.R. (2022). Feedback between a retinoid-related nuclear receptor and the *let-7* microRNAs controls the pace and number of molting cycles in *C. elegans*. *eLife 11*, e80010.

Riddle, D.L., Swanson, M.M., and Albert, P.S. (1981). Interacting genes in nematode dauer larva formation. *Nature 290*, 668-671.

Ragle, J.M., Aita, A.L., Morrison, K.N., Martinez-Mendez, R., Saeger, H.N., Ashley, G.A., Johnson, L.C., Schubert, K.A., Shakes, D.C., and Ward, J.D. (2020). The conserved molting/circadian rhythm regulator NHR-23/NR1F1 serves as an essential co-regulator of *C. elegans* spermatogenesis. *Development 147*, dev193862.

Reinhart, B.J., Slack, F.J., Basson, M., Pasquinelli, A.E., Bettinger, J.C., Rougvie, A.E., Horvitz, H.R., and Ruvkun, G. (2000). The 21-nucleotide *let-7* RNA regulates developmental timing in *Caenorhabditis elegans*. *Nature 403*, 901–906.

Tsai, K.L., Sun, Y.J., Huang, C.Y., Yang, J.Y., Hung, M.C., Hsiao, C.D. (2007) Crystal structure of the human FOXO3a-DBD/DNA complex suggests the effects of post-translational modification. *Nucleic Acids Res*. 35(20): 6984-94.

Zhang, L., Cheng, J., Ward, J., and Dernburg, A. (2015). The auxin-inducible degradation (AID) system enables versatile conditional protein depletion in *C. elegans*. *Development 142*, 4374–4384.
